# Nanoparticle-Based Drug Delivery Systems in Inhaled Therapy: Improving Respiratory Medicine

**DOI:** 10.3390/ph17081059

**Published:** 2024-08-12

**Authors:** Elena Cojocaru, Ovidiu Rusalim Petriș, Cristian Cojocaru

**Affiliations:** 1 Morpho-Functional Sciences II Department, Faculty of Medicine, “Grigore T. Popa” University of Medicine and Pharmacy, 700115 Iasi, Romania; elena.cojocaruu@umfiasi.ro; 2 Medical II Department, Faculty of Medicine, “Grigore T. Popa” University of Medicine and Pharmacy, 700115 Iasi, Romania; 3 Medical III Department, Faculty of Medicine, “Grigore T. Popa” University of Medicine and Pharmacy, 700115 Iasi, Romania; cristian.cojocaru@umfiasi.ro

**Keywords:** nanoparticles, inhalation, therapy, applications

## Abstract

Inhaled nanoparticle (NP) therapy poses intricate challenges in clinical and pharmacodynamic realms. Recent strides have revolutionized NP technology by enabling the incorporation of diverse molecules, thus circumventing systemic clearance mechanisms and enhancing drug effectiveness while mitigating systemic side effects. Despite the established success of systemic NP delivery in oncology and other disciplines, the exploration of inhaled NP therapies remains relatively nascent. NPs loaded with bronchodilators or anti-inflammatory agents exhibit promising potential for precise distribution throughout the bronchial tree, offering targeted treatment for respiratory diseases. This article conducts a comprehensive review of NP applications in respiratory medicine, highlighting their merits, ranging from heightened stability to exacting lung-specific delivery. It also explores cutting-edge technologies optimizing NP-loaded aerosol systems, complemented by insights gleaned from clinical trials. Furthermore, the review examines the current challenges and future prospects in NP-based therapies. By synthesizing current data and perspectives, the article underscores the transformative promise of NP-mediated drug delivery in addressing chronic conditions such as chronic obstructive pulmonary disease, a pressing global health concern ranked third in mortality rates. This overview illuminates the evolving landscape of NP inhalation therapies, presenting optimistic avenues for advancing respiratory medicine and improving patient outcomes.

## 1. Introduction

Respiratory disorders, including asthma, chronic obstructive pulmonary disease (COPD), and various infections, pose significant challenges to global health. With 454.6 million cases worldwide, chronic respiratory disorders accounted for 4.0 million deaths in 2019 and were the third most common cause of death [1]. Traditional therapies, though effective to some extent, often face limitations such as systemic side effects, poor bioavailability, and suboptimal drug targeting. Since Richard Adolf Zsigmondy won the Nobel Prize in Chemistry in 1925 for his pioneering work on colloids—a particular kind of nanoparticle (NP)—NPs have gained significant attention [2]. But in the last few decades the field of study has seen remarkable growth and development, leading to their widespread use in a range of industries and significant potential in medical applications. Global regulatory entities have been actively involved in evaluating and certifying NPs for a variety of applications. Although the exact number of NP-based products approved by regulatory agencies is unclear, significant progress has been made in this area. Regulatory bodies such as the World Health Organization and the US Food and Drug Administration (FDA) have implemented stringent guidelines to ensure the safe and effective use of NPs in medical applications. These regulations play a crucial role in balancing innovation with patient safety, fostering the development of novel NP-based therapies while mitigating potential risks [3,4]. The FDA defines nanotechnology as the manipulation of materials between 1 and 100 nm (0.001–0.1 μm) in size [5]. Also, the European Union has established guidelines and regulations concerning the use of NPs [6]. These efforts underscore a continued commitment to integrating nanotechnology into consumer and medical products while upholding stringent safety standards. Robust laws and regulations are essential to guarantee the safe and responsible application of NPs and nanomaterials across various industries, including medicine.

In recent years, significant strides have been made in NP-based drug delivery systems (DDS) tailored for respiratory diseases. Innovative delivery systems, including nebulizers, pressurized metered-dose inhalers (pMDIs), soft-mist inhalers (SMIs), and dry powder inhalers (DPIs), have been engineered to optimize drug delivery efficiency. These systems offer advantages such as improved drug stability, controlled release kinetics, and enhanced lung deposition, addressing critical limitations of traditional inhalation therapies [7,8,9,10].

NP-based DDS represent a significant advancement in respiratory medicine, offering a transformative approach to the treatment of chronic and acute respiratory diseases. By addressing the limitations of traditional therapies, these innovative systems hold the potential to revolutionize the management of respiratory conditions, ultimately improving patient care and quality of life [10].

By synthesizing evidence from key academic databases, including Scopus, PubMed, Google Scholar, and Web of Science, this review seeks to underscore the transformative potential of NP-DDS in improving therapeutic outcomes and advancing respiratory healthcare, with a specific focus on the terms “nanoparticles”, “inhalation”, “therapy”, and “applications”.

## 2. NP Inhaled Therapies: Key Advantages

Inhaled therapies are crucial for treating respiratory disorders due to the extensive surface area of the lungs, totaling approximately 140 m^2^ at the alveolar level [11]. This anatomical feature ensures direct access to a dense vascular network, highlighting the necessity for effective DDS that target specific lung regions while minimizing systemic side effects through dose reduction [12]. NPs, typically sized under 1 micrometer and often within the nanometer range, play a crucial role in achieving these objectives. Their small size facilitates deep lung penetration, enhancing drug absorption, bioavailability, solubility, and diffusion kinetics [13].

Recent systematic reviews emphasize the efficacy of NP inhalation therapies, which achieve higher drug deposition in the lungs than conventional methods [12,13,14]. This targeted approach is particularly advantageous for diseases necessitating precise drug localization, such as respiratory infections and localized inflammation.

Between 12% and 40% of the administered dose reaches the lungs, with approximately 20% to 25% of the medication remaining within the inhalation device [15,16,17], underscoring ongoing optimization opportunities. Smaller drug dimensions facilitate deeper penetration into peripheral and distal lung regions, as larger particles (>10 µm) are confined to larger airways. Particle size optimization is critical: particles sized between 1–5 µm target small airways effectively [18,19], while those under 500 nm reach alveolar sites via Brownian diffusion [20]. NPs smaller than 200 nm demonstrate effective targeting of respiratory epithelial cells while evading macrophage clearance and rapid cellular uptake, essential for sustained therapeutic efficacy [21,22].

The mucus layer, with pore sizes ranging from 100 to 500 nm, selectively traps aerosols based on size due to its adhesive properties. However, particles smaller than 100 nm may migrate toward central airways and potentially exit the respiratory tract [23].

Furthermore, NPs serve as efficient carriers for therapeutic molecules such as micro RNAs (miRNAs), showing promise in treating diverse respiratory disorders, including cancers, ischemic stroke, and pulmonary fibrosis [24]. Upon reaching target areas, effective aerosol behavior involves evading local elimination mechanisms such as sneezing, swallowing, mucociliary clearance, surfactant layers, and macrophage phagocytosis at the alveolar level [25].

When designing nanoparticles for inhalation, several crucial factors must be considered to ensure both their effectiveness and safety (Table 1) [26]. First, biocompatibility is essential, as the nanoparticles need to interact safely with biological systems. They should also be biodegradable so they break down naturally without causing harm. Efficient cellular uptake is important for their effectiveness in entering cells, while controlled release mechanisms allow for precise drug delivery over time. Cost-effectiveness is key for scaling up production and ensuring economic viability. Understanding the dose response and maintaining dose uniformity is important for assessing how the administered dose impacts biological responses and ensuring consistent drug delivery. The capacity for drug loading affects how much drug can be incorporated into the nanoparticles, which impacts their therapeutic effectiveness. It’s also crucial to consider the immune response to prevent unwanted immune reactions. The mass median aerodynamic diameter is significant for ensuring that nanoparticles reach the lungs effectively, with the fine particle fraction being vital for penetrating deeper lung regions. Pharmacokinetics involves studying the absorption, distribution, metabolism, and excretion of nanoparticles within the body. Efficient production processes are necessary for cost-effective manufacturing, while safety involves evaluating potential toxicity and health risks. Sustainable production practices are important for minimizing environmental impact, and mucosal penetration is critical for effective drug delivery across mucosal barriers. Entrapment efficiency reflects how well the drug is encapsulated within the nanoparticles, and ensuring stability during airflow is crucial. Reducing the rate of nanoparticle clearance from the body and optimizing lung deposition enhance the effectiveness of drug delivery, while a sustained release profile ensures long-lasting therapeutic effects.

Ongoing innovations include surface modifications to enhance mucus penetration and magnetic guidance systems for precise lung targeting [27].

**Figure 1:**
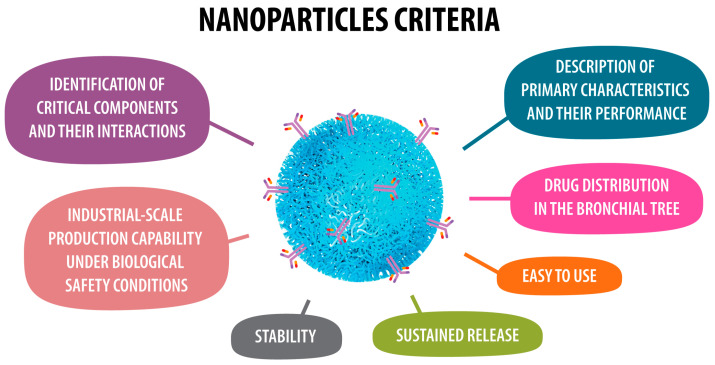
Nanoparticle characteristics.

Figure 1 highlights key characteristics of nanoparticles that are vital for their application and performance.

Chemical stability ensures nanoparticles remain unchanged under different conditions. Dispersibility refers to their ability to spread evenly in airflow. Physical stability maintains their structure over time, avoiding aggregation or degradation. The shape and size, measured in nanometers, impact their behavior and effectiveness. Surface charge affects interactions with other substances, while surface functionalization enhances targeting capabilities. Particle-particle interactions influence stability and dispersion. Lastly, low inertia is crucial for applications involving airflow, ensuring nanoparticles follow airflow patterns effectively.

## 3. Advances in Technology and Delivery Systems

Since their introduction in clinical trials in the early 1990s [28], NPs have been explored extensively for their potential in targeted drug delivery to the lungs, addressing challenges such as limited bioavailability and systemic side effects. Two primary methods are employed for delivering NPs to the lungs: aerosolization of solid microparticles utilizing a Trojan Horse strategy and nebulization of nanosuspensions dispersed in micron-sized droplets via nebulizers [29,30]. Studies suggest that approximately 50% of nebulized substances are effectively inhaled, highlighting ongoing optimization needs [31].

### 3.1. Advances in Types of NPs Used

**Figure 2:**
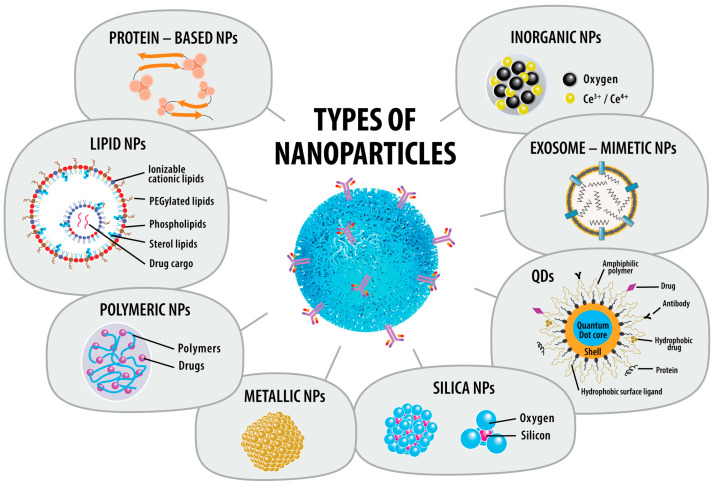
Types of nanoparticles. Legend: NPs—nanoparticles; QDs—quantum dots.

Most inhaled NPs predominantly accumulate and release slowly in the alveolar region. Recent studies in NP-based inhalation therapies aim to minimize side effects, enhance therapeutic effectiveness, and optimize lung-specific drug delivery. Figure 2 presents some of the latest and most promising NPs explored for inhalation therapy.

By detailing the different types of carriers used for these nanoparticles, elucidating their mechanisms of action, and listing the available drugs, Table 2 aims to provide an organized reference for understanding the current landscape of inhaled nanoparticles.

#### 3.1.1. Lipid-NPs (LNPs)

LNPs have emerged as versatile carriers in inhalation therapy, leveraging their unique structure and properties to enhance drug delivery efficiency and therapeutic outcomes in respiratory medicine. LNPs consist of various components, including ionizable lipids, solid and liquid lipids, and surfactants that play a crucial role in facilitating endosomal membrane binding and subsequent cytosolic release of encapsulated therapeutic agents [32]. Also, cholesterol and polyethylene glycol-coated lipids are essential for complexing mRNA and facilitating in vivo delivery [63]. This lipid shell not only provides structural integrity but also ensures stability in aqueous environments, which is crucial for the protection and controlled release of encapsulated drugs within the respiratory tract.

In addition, LNPs can be functionalized with targeting ligands, such as peptides or antibodies, allowing specific recognition and binding to receptors on lung epithelial cells or affected tissues, thereby enhancing therapeutic efficacy and minimizing off-target effects [34].

LNPs offer a promising method for administering genetic therapies directly to lung tissues, presenting potential treatments for genetic conditions such as cystic fibrosis (CF) through gene correction. Research has shown that LNPs can be customized for efficient mRNA delivery to the lungs, emphasizing the necessity for stability during nebulization and effective penetration through mucus [64].

In oncology, LNPs play a crucial role in targeted drug delivery for lung cancer. By encapsulating chemotherapeutic agents or gene therapies, LNPs enable precise localization within lung tumors, enhancing therapeutic efficacy while minimizing systemic toxicity and adverse effects [65,66].

Furthermore, LNPs loaded with anti-inflammatory drugs or antibiotics hold promise for treating inflammatory lung diseases by delivering drugs directly to affected areas, thereby improving patient outcomes and quality of life [66,67].

Curcumin’s anti-lung cancer effects are attributed to its potent antioxidant and anti-inflammatory properties, as well as its ability to enhance apoptosis [68] (Table 2). Inhalation delivery enhances topotecan exposure to lung tissue and improves its effectiveness against lung cancer [35]. Celecoxib was encapsulated in nanostructured lipid carriers, and their lung deposition in mice revealed that these carriers exhibited favorable fine particle fraction and mass median aerodynamic diameter [69]. Fluticasone propionate (FP)-loaded solid LNPs are more effective in managing oxidative stress compared to FP treatment alone [36]. Inhaled delivery of Irinotecan has shown promise, especially as a second-line therapy for small cell lung cancers (SCLCs) [37]. NLD1, which incorporates high levels of PEG-lipid and cationic lipid helper, demonstrates that increasing PEG content in LNPs improves mRNA delivery to the lungs via nebulization [39]. The optimized lipid nanoparticles for nebulized mRNA delivery to the lungs have been shown to be more effective in preventing H1N1 in mice compared to systemic administration [39].

#### 3.1.2. Polymeric NPs (PNPs)

PNPs, constructed from biocompatible and biodegradable polymers like chitosan or poly(lactic-co-glycolic acid) (PLGA), offer significant promise for inhalation therapy. These PNPs have a core made of biodegradable polymers such as PLGA, poly(lactic acid) (PLA), chitosan, alginate, and polycaprolactone (PCL). These materials degrade into non-toxic by-products that are safely eliminated from the body. Surface modifications, similar to those used in LNPs, can be applied to PNPs to enhance targeting capabilities. Endocytosis is the primary mechanism for cellular uptake of polymeric NPs, leading to intra-lysosomal localization of the therapeutic agents [70].

The deposition of PNPs in the lungs is governed by mechanisms such as impaction, sedimentation, interception, and diffusion, each influenced by particle size. Impaction affects larger particles (>5 µm) in the upper respiratory tract, where airflow direction changes abruptly. Sedimentation impacts particles between 1 and 5 µm due to gravity, affecting the bronchi and bronchioles where airflow velocity decreases. Interception occurs when elongated particles or fibers of similar size to the airways follow the airstream closely and contact airway walls, particularly at bifurcations and in the bronchioles. Diffusion, influenced by the random Brownian motion of very small particles (<0.5 µm), predominantly affects the alveolar region where airflow is minimal and distances to airway walls are short [29,39,40,71].

PNPs are generally safe for pulmonary administration due to their biocompatible and biodegradable nature, minimizing the risk of chronic toxicity. The controlled and sustained release of encapsulated drugs can be fine-tuned by altering the polymer composition and molecular weight. PNPs can encapsulate a broad spectrum of therapeutic agents, including small molecules, peptides, proteins, and nucleic acids (such as small interfering RNA- siRNA, short hairpin RNA, and miRNA), protecting them from degradation and enabling targeted delivery to specific lung cells, thereby increasing therapeutic efficacy and reducing systemic side effects [72].

PNPs have diverse applications in inhalation therapy. They are used to administer anti-inflammatory agents to treat chronic respiratory conditions like asthma and COPD [73]. They can encapsulate antibiotics and antimicrobial agents to treat bacterial infections in the lungs, such as those caused by *Pseudomonas aeruginosa* in CF patients [74]. PNPs can deliver chemotherapeutic drugs directly into lung tumors, improving drug concentration at tumor sites while minimizing systemic toxicity [75]. They are also capable of delivering genetic material, such as DNA or RNA, into lung cells for gene therapy applications, providing potential treatments for genetic disorders like CF [76]. By integrating these advanced PNP technologies, inhalation therapies can achieve precise, effective, and safer treatments for various respiratory diseases, showcasing their potential to revolutionize pulmonary medicine.

We outline key characteristics of PNPs that are specifically designed for delivery via inhalation in Table 3.

Chitosan: Known for its positive charge and hydrogen-bonding capability, chitosan exhibits strong mucoadhesive properties [111]. This enables chitosan-based NPs to adhere to mucosal surfaces in the respiratory tract, extending their residence time and allowing for the prolonged release of encapsulated drugs. Inhaled chitosan NPs significantly enhance bioavailability and therapeutic efficacy [40]. Studies highlight chitosan’s ability to enhance cellular uptake via mechanisms such as clathrin-mediated endocytosis and caveolae-mediated endocytosis, which are crucial for delivering therapeutic payloads into target cells [77]. Beyond its adhesive properties, chitosan’s biocompatibility and biodegradability further contribute to its suitability for biomedical applications, particularly in DDS tailored for respiratory diseases.

Gelatin: Gelatin possesses distinct isoelectric points and exhibits amphoteric activity, which enables it to facilitate cellular uptake and interact with mRNA in a cargo-dependent manner, thus facilitating endosomal release [51]. This makes gelatin-based nanomaterials suitable for delivering treatments for conditions like tuberculosis and for protein and mRNA delivery [49,50,51].

PLGA: While PLGA itself lacks strong mucoadhesive properties, surface modifications (e.g., chitosan coating) can enhance this feature. The polymer’s characteristics, such as erosion time and hydrophilic/hydrophobic balance, can be tailored by adjusting the lactide/glycolide ratio and molecular weight. PLGA’s versatility makes it suitable for various applications, including CF, asthma, and COPD treatments [78,83,84,85,86,87,88].

PLA: PLA-NPs degrade through hydrolysis of the ester bonds in their polymer framework. This degradation process is influenced by environmental variables such as pH and temperature, as well as the molecular weight of the polymer [88]. PLA-NPs are noted for their biocompatibility and biodegradability, which are crucial for their application in DDS. PLA-NPs are extensively studied for their ability to encapsulate and deliver a wide range of therapeutic agents effectively. They have been investigated in various biomedical applications, including drug delivery to treat respiratory diseases [88,89]. Their ability to undergo controlled degradation allows for sustained release of encapsulated drugs, which is advantageous for achieving therapeutic efficacy while minimizing adverse effects.

PEtOx: Poly(2-oxazoline) (PEtOx) is rapidly internalized by cells through endocytosis, with its rate adjustable via polymer structure modifications [91,112]. Its hydrophobic nature enhances cellular uptake, dispersing the polymer mainly in vesicular and perinuclear areas post-internalization [113]. PEtOx is noted for its unique physicochemical properties, non-toxicity, and stability [59,60,83,84].

PCL: PCL and PLGA can target the Golgi/endoplasmic reticulum, accumulating in late endosomes and Golgi-associated vesicles [96,114]. PCL’s endocytic pathways make it effective for treating pulmonary inflammatory diseases and pulmonary hypertension [97,98].

PEI: Polyethyleneimine (PEI) NPs adhere to the pulmonary epithelium and are internalized by receptor-mediated endocytosis or macropinocytosis. PEI’s high cationic charge density facilitates cellular absorption, allowing for sustained drug interactions [99]. PEI is used in gene therapy and lung tumor inhibition due to its efficient cellular uptake and endosomal escape capabilities [99,100,101,102].

PS-NPs: Polystyrene NPs (PS-NPs) are highly versatile in DDS due to their stability, ease of functionalization, and biocompatibility. They primarily interact with cells through clathrin-mediated endocytosis and caveolae-mediated endocytosis pathways, facilitating efficient internalization into target cells for drug delivery applications [103]. PS-NPs have emerged as promising candidates for delivering therapeutic agents to specific respiratory tissues [115]. They are particularly effective in treating respiratory diseases such as asthma and COPD, where their size and surface modifications can be tailored to enhance targeting and therapeutic efficacy [73,74,87]. These NPs exhibit high cellular uptake efficiency, making them ideal carriers for a range of drugs, including anti-inflammatory agents, antibiotics, and therapeutic proteins [104,105]. Beyond therapeutic applications, PS-NPs are explored for diagnostic imaging and targeted therapy. Surface modifications with ligands or antibodies enable PS-NPs to bind specifically to disease markers or receptors, facilitating precise imaging of lung tissues and guiding targeted therapeutic interventions [103].

PVA: Polyvinyl alcohol (PVA) plays a crucial role in respiratory DDS by stabilizing medications and forming effective NPs. It enhances cellular uptake through various mechanisms like clathrin-mediated endocytosis, caveolae-mediated endocytosis, and macropinocytosis [116]. PVA-based NPs are used in inhalation therapy to stabilize and deliver drugs such as antibiotics and anti-inflammatory agents directly to the lungs. For example, PVA facilitates the microencapsulation of medications like vinpocetine, enhancing their efficacy when combined with dioctylsulfosuccinate sodium [117]. Additionally, PVA is essential for creating carrier-free dry powder inhalers for drugs such as ibuprofen, ensuring optimal drug release and particle sizes suitable for pulmonary administration. This polymer is particularly effective in treating respiratory infections and chronic conditions like CF [118]. Despite its benefits, the safety of inhaled PVA remains a concern due to potential risks highlighted in inhalation studies, including serious side effects such as pulmonary embolism associated with PVA microparticle embolotherapy interventions [118].

A higher aerosol performance, along with a sustained release profile, was achieved for PLGA nanoparticles loaded with 5% TAS-103 [42] (Table 2). A potential multifunctional polymeric vesicle combining PLGA and PEG has been proposed for delivering COPD medications, such as prednisolone and theophylline [44,119]. Poly(lactic-co-glycolic acid) nanoparticles loaded with α-1-antitrypsin could be a promising formulation for the treatment of respiratory diseases [45]. Gel-BEGF demonstrated increased cellular uptake in EGFR-overexpressing cancer cell lines, showing significant promise for targeted lung cancer treatment [46]. Paclitaxel-loaded expansile nanoparticles enhanced overall survival in the Lewis lung carcinoma model [120]. Lomustine, a potent antineoplastic agent against the lung cancer cell line L132 showed excellent control of drug release [121]. Camptothecin-loaded poly(glycerolsuccinic acid) dendrimers were investigated as a potential carrier, leading to enhanced cytotoxicity against NSCLC (NCIH460) cells [122]. The novel peptide-dendrimer conjugate as a drug carrier was efficiently taken up by a lung tumor-bearing athymic mouse model [123]. Doxorubicin-loaded dendrimers enhanced anticancer efficacy in a lung metastasis model [124]. A similar approach was utilized to develop a DPI formulation by incorporating doxorubicin-loaded nanoparticles into an inhalable carrier, where doxorubicin was incorporated into poly(butylcyanoacrylate) nanoparticles using emulsion techniques and coated with both polysorbate 80 and dextran [47]. Inhaled cisplatin dry powder has demonstrated activity against lung tumors in mice, inducing upregulation of PD-L1 on these tumors and enhancing the efficacy of anti-PD1 therapy in vivo by promoting the intratumoral recruitment of dendritic cells and T cells [125]. Polyethylene glycol-modified polylactic acid nanoparticles loaded with taxanes enhanced chemoradiation therapy both in vitro and in an A549 lung tumor xenograft model [126].

#### 3.1.3. Metallic NPs (MNPs)

MNPs comprise metals tailored for specific applications in inhalation therapy. MNPs typically consist of a core of metal or metal oxide—like gold, silver, iron, or zinc—enhanced with surface modifications such as functional groups, polymers, or biological molecules (peptides or antibodies) to optimize stability, biocompatibility, and target specificity. MNPs exhibit unique properties beneficial for respiratory medicine, including cell wall interaction, membrane penetration, reactive oxygen species production, DNA damage, and inhibition of protein synthesis. These characteristics make MNPs promising candidates for developing novel antibacterial agents effective against antibiotic-resistant pathogens. For example, aerosolized gold NPs administered via endotracheal delivery in animal models demonstrate potential for systemic drug delivery, minimizing off-target effects associated with repeated cytotoxic therapies [127].

However, inhaling MNPs carries inherent health risks. Research indicates that exposure to metal oxide NPs, including zinc, copper, nickel, and magnesium, can exacerbate allergies by releasing metal ions and inducing inflammation [128]. Additionally, NPs derived from combustion sources such as diesel exhaust particles can cause oxidative stress and inflammation in the lungs, worsening cardiovascular and respiratory conditions [129]. High concentrations of metal oxide fumes, such as those from activities like gunfire, have been linked to lung cell damage, inflammation, immune activation, and systemic effects such as platelet activation and blood coagulation [130].

In respiratory medicine, magnetic MNPs offer versatile applications due to their small size and high surface area-to-volume ratio. These properties make them effective carriers for drug delivery, enabling the overcoming of biological barriers within the respiratory system to enhance therapeutic efficacy [131]. Specifically, inhaled MNPs show great promise in combating multidrug-resistant organisms in lower respiratory tract infections. Additionally, the unique characteristics of MNPs facilitate their use in personalized and targeted drug delivery strategies for various respiratory disorders, including asthma, COPD, pulmonary fibrosis, lung cancer, and lung infections [132].

Moreover, MNPs like silver, gold, and copper exhibit notable antiviral properties, including against viruses such as SARS-CoV-2. This makes them valuable components in nanomedicine-based drug delivery systems aimed at targeted therapy for respiratory diseases, including COVID-19 [133]. Cisplatin-loaded magnetic nanoparticles increased the cytotoxicity of cisplatin in a cisplatin-resistant A549 lung tumor xenograft model [134].

Methotrexate-loaded gold nanoparticles demonstrated increased tumor retention and improved therapeutic efficacy in a Lewis lung carcinoma model [135]. Quercetin-loaded Fe₃O₄ magnetic nanoparticles coated with PLGA are well-suited for targeting lung cancer cells through nebulization [136].

#### 3.1.4. Silica NPs

Silica NPs are abundant on earth and offer controllable particle sizes, large surface areas, and excellent biocompatibility [137]. Their optimal designs and electronic properties have been computationally investigated, highlighting the impact of functionalization on stability and reactivity [138]. Silica NPs possess a high specific surface area, adjustable pore structure, and easy surface modification capabilities [139]. In biomedical applications, silica NPs serve as carriers for anti-inflammatory drugs, enabling localized delivery of high drug doses with minimized side effects and improved biodistribution [140]. Loaded with therapeutic drugs like dexamethasone, they effectively treat airway inflammation when administered via inhalation [141]. Their ability to enhance dissolution and intracellular drug release makes them suitable for dispersed liquid drop dosage forms and dry powder inhalers, which are crucial for pulmonary drug delivery [55]. Silica-modified NPs are also used in inhalation chemotherapy for lung diseases, maintaining high drug concentrations in the lungs and showing promise in disease therapy [142]. Additionally, they are employed with antimicrobial agents to combat antimicrobial resistance and biofilm development, offering solutions to enhance treatment outcomes [56]. Silica NPs have demonstrated potential in delivering genetic material such as siRNA for treating genetic disorders like CF [143,144] and targeting KRAS-mutant lung cancer [145].

Inhaled mesoporous silica nanoparticles were used for targeted drug delivery to the lungs, showing a reduction in TNF-α release [146] (Table 2). Research shows that SiO₂ particles can disrupt EGFR signaling in lung epithelial cells, affecting drug uptake, while ultrasmall core-shell silica nanoparticles enhance the delivery and efficacy of anticancer drugs like gefitinib, leading to reduced tumor growth and extended survival in non-small cell lung cancer models [147,148].

#### 3.1.5. Quantum Dots (QDs)

QDs used in pulmonary applications feature varied compositions, often enhanced with surface-modifying chemicals like thiol, alkoxy, and cycloalkyl groups to optimize their imaging and drug delivery capabilities [149]. These QDs are further coated with specific oligomers or polymers via tailored chemical reactions to enhance tissue targeting and traceability within the body. QDs possess unique optical and electronic properties, coupled with chemically active surfaces, ideal for advanced imaging and precise drug delivery applications [150,151]. They enable optical tracing for diagnosing lung diseases, detecting microbial infections, and identifying lung cancer when administered via inhalation [57,150]. Modified QDs, decorated with ligands such as folic acid, have shown promise as targeted DDS for directing therapeutics like doxorubicin to specific receptors on human adenocarcinoma basal alveolar epithelial cells [152]. In biomedical research, QDs are versatile tools utilized for cell tracking, intracellular delivery, immunolabeling, gene delivery, cellular imaging, and motility assays [153,154]. Their capabilities extend to targeting the nucleus for diagnostic purposes and achieving high sensitivity for miRNA targeting, underscoring their potential in cutting-edge biomedical applications.

Pulmonary delivery of EGFR (Epidermal Growth Factor Receptor)-targeted SPIO (Superparamagnetic Iron Oxide) nanoparticles improved tumor retention and significantly inhibited lung tumor growth through hyperthermic destruction of NSCLC [155] (Table 2). Inhaled quantum dots, such as CdSe and CdTe, are explored for managing lung diseases like microbial infections and lung cancer and targeting alveolar macrophages [150].

#### 3.1.6. Protein-Based NPs (PBNPs)

PBNPs utilize proteins as their primary structural component, forming well-defined hollow structures such as virus capsids, ferritin, and chaperonins through self-assembly [58,156,157]. These NPs offer unique advantages, including excellent receptor selectivity, biocompatibility, and biodegradability [58,158]. PBNPs are suitable for drug delivery applications due to their optimal size for endocytosis, low toxicity, predictable activity profiles, and the ability to functionalize at various interfaces [58,159]. They are particularly promising for incorporating therapeutic enzymes like proteases and endosomal escape enzymes, known for their specificity and efficacy in treating diseases [160].

In respiratory medicine, PBNPs made from proteins like albumin, gelatin, and soy protein are administered via inhalation for conditions such as asthma, COPD, and acute respiratory distress syndrome [59]. These proteins facilitate drug delivery with minimal immunogenicity and allow selective modification of NPs with tumor-specific ligands for targeted therapy [161,162]. For instance, incorporating antibiotics such as gentamicin and ciprofloxacin into soy protein isolate arrays enhances targeted drug delivery, protecting drugs from degradation and enabling controlled release [131,163]. PBNPs also play a role in anticancer therapy, with FDA-approved inhaled protein biologics like Pulmozyme demonstrating their efficacy [164]. Moreover, advancements in protein engineering have led to PBNPs designed for gene therapy applications using modular protein engineering and virus-like particles [165]. However, a challenge with using proteins as drug carriers is their susceptibility to degradation from temperature fluctuations and shear stress during manufacturing or storage [166].

Inhaled paclitaxel-loaded bovine serum albumin microparticle dry powders demonstrated selective interaction with adenocarcinoma cells and significant tumor growth inhibition in a mouse model of lung cancer [167] (Table 2). A nano-drug delivery system using targeting ligand-modified albumin demonstrated enhanced antitumor efficacy against tumor growth and lung metastasis in mice, underscoring the potential of inhaled protein-based formulations for effective cancer treatment [168].

#### 3.1.7. Inorganic NPs

Inhaled inorganic NPs encompass tiny particles of substances like cerium oxide (CNPs) and calcium phosphate, each offering distinct structural and functional properties [60,61]. CNPs consist of cerium atoms in two oxidation states (Ce3+ and Ce4+), exhibiting magnetic, electronic, and multi-enzyme mimetic activities that combat intracellular reactive oxygen species [169]. Calcium phosphate NPs, including hydroxyapatite and tricalcium phosphate, are noted for their biocompatibility, biodegradability, pH-dependent solubility, and drug and nucleic acid (plasmid DNA, siRNA, miRNA, etc.) binding capabilities, making them promising for drug delivery applications [170].

CNPs find utility in cancer therapy due to their ability to induce oxidative stress and damage in lung cancer cells [171]. Surface modification with amorphous silica aims to mitigate potential toxicity, enhancing safety profiles [172]. Studies have shown that CNPs coated with microRNA-146a can protect against acute lung injury induced by bleomycin, reducing inflammation and oxidative stress while preserving lung function [173]. Inhaled cerium phosphate nanoparticles play a significant role in the lungs by precipitating in phagolysosomal regions within macrophages (Table 2) [174].

#### 3.1.8. Exosome-Mimetic NPs (EMNPs)

EMNPs are synthetic structures designed to replicate the natural functions and structure of exosomes, small vesicles crucial for intercellular communication and cargo delivery in cells. These EMNPs are typically fabricated through extrusion, artificial synthesis, or liposome fusion, incorporating proteins, lipids, and nucleic acids found in natural exosomes such as messenger RNAs, noncoding RNAs, and DNA [175,176].

EMNPs offer several advantages in drug delivery. Their lipid bilayer structure protects therapeutic compounds from degradation, ensuring stability and sustained release at the target site. They exhibit high biocompatibility and low immunogenicity, minimizing the risk of adverse immune reactions and enabling precise targeting capabilities. Compared to natural exosomes, EMNPs are more stable, maintaining integrity and functionality during administration and storage [177].

In respiratory medicine, inhaled EMNPs hold significant promise. They have demonstrated anticancer effects by encapsulating chemotherapeutic drugs like paclitaxel within T cell-derived exosomes modified with chimeric antigen receptors, effectively targeting specific cancer cells and reducing tumor growth [178]. Studies in mice have shown that lung-derived extracellular vesicles, when inhaled, distribute mRNA and protein cargo effectively in lung tissues, enhancing bioavailability and therapeutic efficacy [178]. Additionally, inhalable virus-like EMNPs decorated with the SARS-CoV-2 receptor binding domain are being explored as potential COVID-19 vaccine candidates, triggering robust immune responses in animal models and showing stability at room temperature [62]. EMNPs can also carry anti-inflammatory agents for treating lung diseases characterized by inflammation, as well as anti-fibrotic agents to reduce fibrosis and scarring in pulmonary tissues, potentially halting the progression of conditions like pulmonary fibrosis [179,180].

Exosomes have been developed for the delivery of Curcumin to treat lung and breast cancer (Table 2) [181]. F-EMNs loaded with RNAs are utilized in lung disease therapy to enhance targeted delivery of RNA therapeutics and improve therapeutic outcomes through precise gene modulation [182].

#### 3.1.9. Nanocrystals/Nanosuspensions

Nanocrystals are partially amorphous pharmaceutical structures with sizes ranging from 100 to 1000 nm, formed through molecular aggregation. They are designed to enhance solubility, adhesiveness, and distribution in the respiratory tract [183]. Additionally, the large surface area of nanocrystals allows for better absorption and prolonged residence time in the respiratory tract [184].

Suspended nanocrystals of budesonide have been utilized for nebulized treatment of cholesterol-responsive lung diseases [185]. A study comparing the nebulization of budesonide nanocrystals with conventional budesonide formulations revealed that the nebulization time for nanoparticles was shorter, with a similar safety profile and pulmonary absorption rate [46]. Another study investigating the use of itraconazole nanosuspensions in patients with cystic fibrosis found that high concentrations were achieved and persisted for a long time in lung tissue [186]. However, the inhalation administration of nanocrystals/nanosuspensions has not yet been approved for routine clinical practice.

### 3.2. Advances in Delivery Systems

Despite the promising potential of nanomolecules for inhalation therapy, relatively few studies have been successfully completed. Challenges such as the difficulty in obtaining nanoparticles and achieving consistent and reproducible airway releases have slowed progress. Currently, research has largely focused on identifying the most effective carriers for inhalation therapy. However, given the rapid advancements in respiratory disease research, we anticipate an increase in studies showing superior results compared to traditional molecule administration methods. Moreover, we expect that gene therapy using inhaled nanoparticles will become a significant breakthrough, especially for treating conditions like cystic fibrosis and alpha-1 antitrypsin deficiency. The safety and efficacy of administering small, precise doses will be a major advantage in this emerging field.

Advanced techniques for inhaled NP delivery systems (INPDS) represent a significant advancement in overcoming biological barriers and controlling drug release. Despite the unique challenges of the pulmonary route, it offers promising opportunities for noninvasive drug administration. Defined by the National Institutes of Health as medical devices for controlled release or targeted delivery, these systems typically involve biocompatible coatings that protect drugs from degradation [187,188]. INPDS provides several advantages, including precise targeting of drugs to the lungs, enhanced drug absorption and bioavailability, and reduced systemic exposure and side effects. They can encapsulate a wide range of therapeutic agents and ensure controlled and sustained drug release, which is crucial for protecting sensitive medications. Their non-invasive nature enhances patient compliance, and they can serve multifunctional roles by combining therapeutic and diagnostic functions. Moreover, these systems effectively navigate biological barriers like mucus, making them highly effective in treating respiratory diseases and potentially other medical conditions [188]. Classification of INPDS considers functionality, design, intended application, and patient-related factors, ensuring tailored optimization to meet specific therapeutic needs and regulatory standards.

#### 3.2.1. DPIs 

DPIs deliver medication in a powdered form that becomes aerosolized upon inhalation, making them increasingly popular for NP delivery due to their user-friendly design, portability, and stability [189]. Ongoing research focuses on optimizing DPI formulations containing NPs by enhancing aerodynamic properties and ensuring redispersibility. Strategies like cryo-milling have been investigated to maintain drug stability and improve inhalation characteristics, demonstrating successful preservation of enzymatic activity even after extended storage periods [190]. The effectiveness of DPI formulations relies on critical factors such as particle size, shape, and surface area, which significantly impact aerodynamic properties and deposition efficiency in the lungs. To expedite the translation of NP-based DPI formulations from research to clinical use, addressing gaps in preclinical development and implementing robust bio-fate linking methodologies are crucial steps [191].

#### 3.2.2. MDIs

MDIs use a propellant to deliver a specific dose of medication as a mist. To study the pulmonary delivery process and therapeutic outcome, a variety of DDS, including lipid-based nanocarriers [192,193], dendrimers, and polymeric nanocarriers [194], have been integrated with MDI. Also, PNPs play a significant role in DDS based on metered dose inhalation [195,196].

MDIs are crucial devices for administering medication for asthma and COPD, utilizing compressed propellants to generate aerosols [197]. These handheld devices can deliver precise drug doses directly or via attachments such as spacers and valved holding chambers, which improve dose synchronization and facilitate inhalation over multiple breaths [197]. Some MDIs incorporate features like one-way valves and deflectable diaphragms to aid in proper inhalation and exhalation processes for patients. Advances include inhalers equipped with processors and memory for monitoring dose parameters and user feedback, enhancing medication consumption effectiveness. NPs significantly enhance the performance and stability of MDIs. Research has explored various NP types, such as lipid nanocarriers, PNPs, dendrimers, and micelles, to effectively deliver pulmonary drugs [198]. An innovative approach involves using anhydrous reverse micelle NPs to mitigate sedimentation instability in peptide-containing pressurized MDIs, resulting in improved particle size, aerosolization properties, and storage stability [199].

#### 3.2.3. Nebulizers

Nebulizers transform liquid medication into a fine mist for inhalation through various types: jet nebulizers utilize compressed air, ultrasonic nebulizers employ ultrasonic waves, and vibrating mesh nebulizers use a vibrating mesh to generate the mist. Nebulizers play a pivotal role in delivering pulmonary medications, offering benefits such as ease of use, applicability across diverse patient groups, and visible spray for patient satisfaction [200]. Recent advancements in particle engineering have facilitated the creation of new inhaled nanosystems that enhance lung deposition and reduce pulmonary clearance, potentially transforming the treatment landscape for severe respiratory conditions like SARS-CoV-2 [200]. Furthermore, innovative techniques such as electro-hydrodynamic nebulizers have emerged to produce and administer polymeric aerosols simultaneously, converting liquid solutions into polymeric particles ideally sized between 1–5 μm for effective lung drug delivery [201]. These innovations underscore the continuous evolution of nebulizer technology for NPs, aiming toward more efficient and targeted delivery of inhaled NPs and promising improved therapeutic outcomes.

#### 3.2.4. SMIs 

SMIs produce a slow-moving, fine mist of medication without the use of propellants. SMIs deliver inhalable drug aerosols, offering benefits such as reduced oropharyngeal deposition and user-friendly operation. The inhalers are poised to advance with NP-based formulations, enhancing targeting precision for specific lung regions and delivering sensitive biologics such as vaccines and proteins [202].

#### 3.2.5. pMDIs

pMDIs are akin to MDIs but specifically tailored for NP formulations. pMDIs that contain NPs are typically formulated to enhance drug delivery efficiency, improve lung deposition, and potentially reduce systemic side effects. Specific examples of pMDI-delivered drugs that can be incorporated into NPs include corticosteroids, bronchodilators, antibiotics [102,203], antibodies [204], and peptides [204]. pMDIs are utilized with various advanced DDS such as lipid nanocarriers, PNPs and dendrimers for pulmonary drug delivery, leading to better therapeutic outcomes [204].

#### 3.2.6. Nasal Sprays 

Nasal sprays deliver NPs through the nasal route, potentially facilitating delivery to the lungs. Nasal NPs are increasingly recognized for their potential to deliver vaccines and antiviral drugs, leveraging the nasal cavity’s permeability and presence of immunocompetent cells [205]. These NPs enhance drug bioavailability, protect against enzymatic degradation, and facilitate drug transport across mucosal barriers. Research indicates their effectiveness in delivering antigens, eliciting robust immune responses without requiring adjuvants [206]. Furthermore, formulations like NanoSTING, encapsulated in liposomes, have demonstrated rapid activation of the innate immune system, offering broad-spectrum protection against respiratory viruses such as SARS-CoV-2 and influenza [206]. Thus, nasal NPs present a promising approach for efficient and targeted delivery of vaccines and antiviral drugs, with promising advantages in efficacy, safety, and ease of administration.

#### 3.2.7. Nasal Nebulizers 

Nasal nebulizers are specialized devices designed to deliver aerosolized NPs through the nasal passages. Nasal nebulizers utilizing NPs show significant potential for enhancing drug delivery efficiency. Current research focuses on developing advanced NP formulations for intranasal use, aiming to surmount biological barriers and enhance drug bioavailability [207,208]. NPs, such as those based on chitosan, protect drugs from degradation, sustain release, and improve mucosal permeation, thereby enhancing the efficacy of vaccines and medications [209]. Additionally, methodologies have been developed to assess NP transport efficiency using nebulizers, providing a valuable tool for evaluating micro-/nanoflow systems in drug delivery [210].

In conclusion, the development and application of various inhaled drug delivery systems for nanoparticles offer promising advancements in respiratory therapy (Table 4).

Lipid, polymeric, metallic, silica, quantum dots, protein-based, inorganic, and exosome-mimetic nanoparticles each present unique advantages for drug delivery. Nebulizers stand out for their broad compatibility with different nanoparticle types despite handling challenges. DPIs offer significant improvements over traditional nebulization in terms of convenience and efficacy, while MDIs and pMDIs provide targeted delivery options. Nasal delivery systems, particularly with quantum dots and metallic nanoparticles, are emerging as valuable tools for localized and systemic treatments. Continued research and optimization of these delivery systems are essential to fully realize the therapeutic potential of nanoparticle-based inhalation therapies, ultimately enhancing patient outcomes in respiratory healthcare.

## 4. Discussion

The practical application of inhaled medications faces numerous challenges that impact both the distribution of inhaled molecules in the bronchial tree and their absorption. Despite these challenges, numerous studies have explored the use of NPs in clinical practice for diagnostic (e.g., imaging) and therapeutic purposes. NPs show promise across various fields, notably in neoplastic pathologies such as lung, breast, and ovarian cancers. In these contexts, NPs are often administered intravenously, with notable examples including doxorubicin liposomes and albumin-bound paclitaxel [211,212]. Additionally, MNPs have demonstrated antimicrobial effects [213].

Medications administered via inhalation encounter specific and nonspecific defense mechanisms that can reduce their bioavailability. However, using NPs as drug carriers offers a way to bypass these protective mechanisms. Studies have shown that NPs can deposit in the lining fluid, protecting them from mucociliary clearance and alveolar macrophages [214]. Major classes of inhaled medications include corticosteroids, bronchodilators (β2 adrenergic and anticholinergic agents), and antibiotics, widely used in treating obstructive bronchopulmonary diseases like asthma and COPD. The recent SARS-CoV-2 pandemic has spurred research into inhalation NPs, with studies evaluating the efficacy and safety of inhaling exosomes in patients with SARS-CoV-2 pneumonia and the impact of inhaling nanosilver on lung immune responses. Current research also investigates the therapeutic effects of inhaling colloidal silver or remdesivir NPs in COVID-19 patients [215]. The success of mRNA in controlling the SARS-CoV-2 pandemic has revealed its potential in other areas. For example, LNPs carrying mRNA are being studied for aerosol delivery to patients with CF. Early results show stable lung function, with forced expiratory volume in the first second remaining stable after treatment, and administration safety has been confirmed [216].

Several molecules are already in clinical use, such as tobramycin, amikacin, and surfactant proteins SP-B and SP-C, utilizing liposomes or distearoylphosphatidylcholine as nanocarriers. Others, like plasmid DNA, are in various development stages [12].

Strong suppression of inflammation in allergic asthma has been achieved in phase I/II studies using interleukin-4 receptor subunit alpha (IL-4Ra) antagonists with lipocalin-1 nanocarriers. Prolonged bronchodilator effects have also been noted with Salbutamol sulfate administered via vesicular/niosomes delivery systems. Studies by Matsuo and Kenyon have shown that incorporating steroids (beclomethasone or dexamethasone) into NPs provides superior anti-inflammatory effects in asthma patients [217,218]. Biocompatible and biodegradable NP forms of beclomethasone (stealth nanosteroids) have reduced eosinophil counts in bronchoalveolar lavage fluid. Encapsulated budesonide in experimental models has similarly reduced inflammation and bronchoconstriction, as measured by eosinophil peroxidase activity, eosinophil counts, and serum immunoglobulin E levels. Notably, weekly administration of liposomal NPs was as effective as daily budesonide therapy, potentially improving adherence and reducing side effects [219]. Similar reductions in inflammatory cells have been observed with NP-containing budesonide therapy [203,220].

The administration of liposome aerosol particles has demonstrated efficient distribution throughout the respiratory tract, sustained for up to 48 h, compared to salbutamol sulfate [221]. This suggests improved and prolonged bronchoconstriction control, with better deposition in the respiratory airways and reduced retention in the oropharynx [222].

Anti-inflammatory effects have been observed in studies using poloxamer 407-based nanoemulsions [223]. For instance, in vitro treatment of human basal epithelial cells sensitized to cigarette smoke resulted in the inactivation of pro-inflammatory cytokines and the activation of anti-inflammatory cytokines, suggesting protective effects on the respiratory mucosa.

Other therapeutic options for managing chronic respiratory diseases involve using small noncoding RNA molecules, such as mature miRNAs [224].

In COPD, miR-146a shows promise for regulating inflammatory cytokines by suppressing IL-1R and Toll-like receptors. Nanotechnologies offer potential solutions for pulmonary antibiotic delivery, with PNPs, especially those based on lactic or glycolic acid, being preferred for respiratory infections due to their biodegradability and lack of toxicity. These advantages have led to the approval of some PNPs, such as chitosan, for practical use. Inorganic nanoparticles like gold NPs and quantum dots also show significant potential [225]. A study by Rathnayake et al. demonstrated that liposome-encapsulated antibiotics release the active substance upon degradation by bacterial lipase in *Pseudomonas aeruginosa* infections, effectively combating both extracellular and intracellular drug-resistant bacteria and offering a positive outlook on antibiotic resistance [226].

For developing inhalation therapies and evaluating device performance, clinical studies utilize various methodologies to estimate drug distribution in the airways. Although in vitro activity does not always correlate with clinical response, understanding lung deposition is crucial. In silico studies, combining imaging with physical models of particle movement generates distribution patterns of inhaled particles. Computational and artificial intelligence-based approaches can predict interactions between inhaled molecules and biological structures, as well as the pharmacokinetic mechanisms involved [227].

An ongoing concern in current therapy is the likelihood of water-soluble nanoparticles causing side effects. Since data on the long-term neutralization and removal of nanoparticles are still limited, there are concerns about potential undesirable effects. Beyond endotoxic effects, there is also significant worry about the environmental impact, prompting the introduction of risk mitigation measures for nanotechnology. Studies aimed at understanding tissue damage caused by the shape or chemical composition of nanoparticles are ongoing and include identifying biomarkers as toxicity sensors.

The interaction of nanoparticles with alveolar macrophages could reduce their efficacy and increase the risk of infection. Therefore, during the synthesis of inhaled nanoparticles, bypassing alveolar macrophages to enhance the availability of administered molecules remains a key goal. Other cellular interactions, such as those with epithelial cells and fibroblasts, also warrant attention. Intracellular uptake of nanoparticles can lead to the generation of oxygen free radicals and the release of cytokines, triggering a pro-inflammatory response [228]. Interaction with fibroblasts can contribute to the development of pulmonary fibrosis, while inflammation and an excess of free radicals may promote the formation of new lesions. The induction of inflammation could counteract anti-inflammatory medications used for conditions like bronchial asthma and COPD, which would be undesirable. Current data suggest that nanoparticles based on silicon, carbon, silver, and zinc may cause adverse respiratory effects [229,230,231]. Most studies are based on experimental models, and little is known about the effects in humans. It has been suggested that nanoparticles may accumulate in the pulmonary alveoli, potentially contributing to the development of pulmonary fibrosis [232]. Due to their small size, nanoparticles are likely to pass through the alveolocapillary membrane, potentially causing systemic effects, particularly depending on their chemical structure.

This article has highlighted key NP types such as LNPs, PNPs, MNPs, silica NPs, QDs, PBNPs, inorganic NPs, and EMNPs used in inhalation therapy. However, numerous other NP varieties and applications exist beyond those discussed, including targeted drug delivery, imaging, diagnostics, gene therapy, anti-microbial and anti-inflammatory effects, and other therapeutic uses.

Researchers are actively exploring innovative NP formulations, surface modifications, and functionalization to enhance biocompatibility and targeting capabilities, addressing specific challenges in diverse medical contexts. This ongoing research underscores the potential of NPs across various domains, reflecting their dynamic role in advancing scientific exploration and innovation.

In conclusion, NPs remain a focal point of significant scientific interest and innovation. Ongoing research efforts aim to enhance their capabilities and broaden their applications, particularly in respiratory medicine.

## 5. Conclusions

NPs are at the forefront of inhalation therapy research, with efforts focused on optimizing design, enhancing safety profiles, and maximizing therapeutic efficacy for various pulmonary diseases. Beyond inhalation therapy, NPs are a vibrant research area spanning medicine, electronics, materials science, and environmental applications.

This article has highlighted key NP types such as LNPs, PNPs, MNPs, silica NPs, QDs, PBNPs, inorganic NPs, and EMNPs used in inhalation therapy. 

This ongoing research underscores the potential of NPs across various domains, reflecting their dynamic role in advancing scientific exploration and innovation.

Several options for inhaled drug delivery are available today, but adapting these methods to nanoparticle therapy necessitates further research. Currently, nebulization devices are the most widely used for inhaled nanoparticle delivery, yet they present challenges due to their size and the labor-intensive handling required. Dry powder inhalers (DPIs) offer significant advantages over nebulization therapy and, for now, are preferred over pressurized metered-dose inhalers (pMDIs) due to their ease of use and improved patient compliance. Ongoing research efforts aim to enhance their capabilities and broaden their applications, particularly in respiratory medicine.

## 6. Challenges and Future Directions

Achieving optimal particle size, surface loading efficiency, and reliable delivery systems is pivotal to ensuring adequate NP concentration at targeted lung sites. However, these goals pose significant challenges. Long-term safety assessments and rigorous regulatory approval processes are imperative, particularly for repeated administration in chronic conditions.

To successfully transition NP-based inhalation treatments from laboratory development to clinical application, scalable and reproducible manufacturing techniques are essential. These efforts are crucial for realizing the full potential of inhaled NP-based therapies in respiratory medicine.

Nanoparticles present exciting opportunities for personalized and gene therapy in conditions like cystic fibrosis. However, implementing nanoparticles in inhalation therapy involves challenges, particularly in incorporating gene therapy into DPIs or pMDIs. Overcoming technical barriers, such as ensuring particle stability, consistent dose delivery, and maintaining low internal resistance for patients with low inspiratory flows, is essential.

These nanoparticles can be designed to target specific cells by integrating corrective genes into cellular DNA. Although these technologies hold revolutionary potential, they will likely provoke ethical debates regarding the extent of genetic manipulation.

For these nanoparticles to be practically and widely used, it is crucial to fully understand their interaction mechanisms within the respiratory tract and the pathogenic pathways affected by the released molecules.

## Figures and Tables

**Table 1:** Inhaled nanoparticles criteria.

Key Criteria	Specifications
Biocompatibility	Compatibility with lipids/ proteins/polymers/metals
Biodegradability	Non-toxic
Cellular uptake	Efficiently taken up by target cells
Controlled release	Triggered release of drugs at the target site
Cost-effectiveness	The economic viability of producing nanoparticles for widespread clinical use
Dose response	Predictable therapeutic response
Dose uniformity	Consistent dosing in each inhalation
Drug loading capacity	High
Immune response	Minimal; reaction of the immune system to the nanoparticles
Immunogenicity	Engineering to avoid triggering a significant immune response
Optimal aerodynamic diameter	1–5 μm for deep lung deposition
Pharmacokinetics	Prolonged retention
Production efficiency	Large-scale production
Safety	Low toxicity
Sustainable production	Environmentally friendly materials
Mucosal penetration	Able to cross mucosal barriers
Mass median Aerodynamic diameter	Important for deposition in Respiratory airways
Fine particle fraction	Nanoparticles small enough to reach deeper regions of the lungs
Protect drug entity in airflow	Able to ensure the stability and integrity of the drug during delivery
Ensure specific site targeting	Precision in nanoparticle delivery into the respiratory tract
Minimizing clearance	Reducing the rate at which the body removes the nanoparticles
Sustained release profile	The ability to release a drug over an extended period

**Table 2:** Inhaled nanoparticle insights.

Inhaled Nanoparticles	Material	Nanoparticle Functional Mechanisms	Nanoparticle and Nanoemulsion Formulations
LNPs	Liposomes solid lipid	Encapsulation [32]	- Liposomal SN-38 [33] - Curcumin [34]
			- TOPOTECAN [35] - Cxb-NLCs [34] - Fluticasone propionate [36]
			- Irinotecan [37] - NanoDEX [38] - NLD1 [39] - Budesonide [34] - LNPs-mRNA [32]
PNPs	- PLGA - Chitosan - PEG	- Mucoadhesion [40] - Enhanced cellular uptake via endocytosis [41] - Protection from enzymatic degradation [34]	TAS-103 [42] NanoDEX [43] Prednisolone [44] Theophylline [44] NLD1 [39] PLGA-α1AT [45]
			Gel-BEGF [46] Paclitaxel [34] Lomustine [34] CPT-PGSA [34] Dox-Pep-Dend [47] PEG-PLA-taxanes NPs [34]
MNPs	- Gold - Silver - Fe_3_O_4_	- Generation of ROS [48] - Disruption of microbial membranes [49] - Targeted delivery via surface conjugation [50] - Enhanced cellular uptake [51]	MTX-AuNPs [52] Cis-MagNPs [53] Q-Fe₃O₄-PLGA-MNPs [54]
Silica NPs	Mesoporous Silica SiO_2_	- Enhanced cellular uptake [51] - Surface functionalization for targeted delivery [55]	Core-shell silica Nanocarriers [56]
QDs	Semiconductor materials (e.g., CdSe, CdTe)	- Fluorescent labeling [57] - Cellular uptake via endocytosis [51]	CdSe/CdTe NPs [57]
PBNPs	Albumin	- Receptor-mediated endocytosis [58] - Enzymatic degradation [58]	NDDS [59] PTX-BSA [59]
Inorganic NPs	- Calcium phosphate - Iron oxide - CeO_2_	- Magnetic targeting [60] - Cellular uptake via endocytosis [51]	CeO_2_ NPs [61]
EMNPs	Lipid bilayers mimicking exosomes	- Drug encapsulation [61] - Fusion with target cell membranes [61] - Intercellular communication [61]	Curcumin [61] F-EMNs [62]

Legend: NPs—nanoparticles; LNPs—lipidic nanoparticles; Cxb-NLCs—celecoxib (Cxb) in nanostructured lipid carriers; nanoDEX—dexamethasone-loaded neutrophil nanoparticles; NLD1-nebulized lung delivery 1; PNPs—polymeric nanoparticles; PLGA (poly(lactic-co-glycolic acid); PEG—polyethylene glycol; TAS-103—(6-[[[2-(dimethylamino)ethyl]amino]carbonyl]-1H-benz[de]isoquinoline-1,3(2H)-dione hydrochloride); PLGA-α1AT -PLGA loaded with α-1—antitrypsin; Gel-BEGF—gelatin nanoparticle system decorated with biotinylated Epidermal Growth Factor; CPT-PGSA—Camptothecin-loaded poly(glycerolsuccinic acid); Dox-Pep-Dend -Doxorubicin-loaded dendrimers novel peptide-dendrimer conjugate; PEG-PLA-taxanes NPs—polyethylene glycol modified polylactic acid nanoparticles loaded with taxanes; MNPs—metallic nanoparticles; Fe_3_O_4_—magnetite, iron oxide; ROS—reactive oxygen species; MTX—Methotrexate; MTX-AuNPs -Methotrexate loaded gold nanoparticles; Cis-MagNPs—Cisplatin-loaded magnetic nanoparticles; Q-Fe₃O₄-PLGA-MNPs—Quercetin-loaded Fe_3_O_4_ magnetic nanoparticles coated with a polymer (PLGA); SiO₂—silicon dioxide; QDs—Quantum dots; CdSe—cadmium selenide; CdTe—cadmium telluride; PBNPs—protein-based nanoparticles; NDDS—nano-drug delivery system; PTX-BSA—paclitaxel (PTX) loaded bovine serum albumin (BSA); CeO_2_ -cerium oxide; EMNPs—Exosome mimetic nanoparticles; F-EMNs (fluorescent-engineered mimetic nanoparticles) loaded with RNAs.

**Table 3:** Characteristics of inhaled polymeric NPs.

Polymeric NPs	Mechanism of Action (after Degradation following Inhaled Administration)	Respiratory Applications
Chitosan	- Mucoadhesion - Cellular uptake (clathrin-mediated endocytosis and caveolae-mediated endocytosis) [77]	Asthma [78] Lung cancer [79]
	- Endosomal escape (“proton sponge effect”) [80] - Intracytoplasmic release of encapsulated therapeutic agents	
Gelatin	- Cellular uptake - Endosomal escape through “the proton sponge effect” [41]	Tuberculosis [81] Protein and mRNA delivery [41]
		Anti-tumoral [82]
PLGA	- Mucoadhesion (surface modifications; e.g., coating with Chitosan) - Cellular uptake (clathrin-mediated endocytosis, fluid-phase pinocytosis) [83] - Endosomal escape through “the proton sponge effect”	CF [84] Asthma [78] Tuberculosis [85] COPD [86] Anti-tumoral [87] Drug delivery [88]
PLA	- Hydrolysis [88] - Endocytosis - Endosomal Escape (“proton sponge effect” or direct fusion with the endosomal membrane) [88]	Drug delivery [88,89]
PEtOx	- Endocytosis [90,91]	Anti-inflammatory [92]
		Respiratory infections [93]
PCL	- Cellular uptake (clathrin-mediated endocytosis and caveolae-mediated endocytosis) [94] - Micropinocytosis [95] - Late endosome [96]	Pulmonary inflammatory diseases [97] Pulmonary hypertension [98]
PEI	- Cellular uptake (endosomal and non-endosomal) - Endosomal escape- “proton sponge effect” [80] - Nuclear uptake organelle disturbance [99]	Gene therapy [99,100] Lung tumor inhibition [101,102]
PS-NPs	- Cellular uptake (clathrin-mediated endocytosis and caveolae-mediated endocytosis) [103]	Drug delivery [104,105]
PVA	- Cellular uptake (clathrin-mediated endocytosis and caveolae-mediated endocytosis) - Macropinocytosis [106]	Respiratory infections [107,108] Smoke filtration [109] Nasal drug delivery [110]

Legend: NPs—nanoparticles, mRNA—messenger RNA, PLGA—poly(lactic-co-glycolic acid), CF—cystic fibrosis, COPD—chronic obstructive pulmonary disease, PLA—poly (lactic acid), PEtOx—poly(2-ethyl-2-oxazoline), PCL—polycaprolactone, PEI—polyethyleneimine, PS—polystyrene, PVA—polyvinyl alcohol.

**Table 4:** Nanoparticle-based delivery systems.

	LNPs	PNPs	MNPs	Silica NPs	QDs	PBNPs	Inorganic NPs	EMNPs
**DPIs**	**+**	**+**	**+**	**+**				
**MDIs**	**+**	**+**		**+**				
**nebulizers**	**+**	**+**	**+**	**+**		**+**	**+**	**+**
**SMIs**								
**pMDIs**	**+**	**+**		**+**				
**nasal sprays**					**+**			
**nasal nebulizers**				**+**				

Legend: LNPs—lipid nanoparticles; PNPs—polymeric nanoparticles; MNPs—metallic nanoparticles; QDs—quantum dots; PBNPs—protein-based nanoparticles; EMNPs—exosome-mimetic nanoparticles; DPIs—dry powder inhalers; MDIs—metered-dose inhalers; SMIs—soft-mist inhalers; pMDIs—pressurized metered-dose inhalers; “+”—indicates the applicability.

## Data Availability

Data sharing is not applicable.
